# Hepatitis C virus genotypes in Saudi Arabia: a future prediction and laboratory profile

**DOI:** 10.1186/s12985-017-0873-7

**Published:** 2017-11-02

**Authors:** Amen Bawazir, Fahad AlGusheri, Hoda Jradi, Mohammed AlBalwi, Abdel-Galil Abdel-Gader

**Affiliations:** 1 The King Abdullah International Medical Research Center (KAIMRC), Community and environmental Health,College of Public Health & Health Informatics. King Saud Bin Abdulaziz University for Health Sciences, Riyadh, 11481 Saudi Arabia; 2 Division of Molecular Pathology and Genetics, Department of Pathology and Laboratory Medicine, King Abdulaziz Medical City, Ministry of National Guard Health Affairs, Riyadh, Saudi Arabia; 3 College of Medicine, King Saud bin Abdulaziz University for Health Sciences, King Abdulaziz Medical City, Ministry of National Guard Health Affairs, 3660, Riyadh, 11481 Saudi Arabia; 4 Department of Basic Medical Sciences, College of Medicine, King Saud bin Abdulaziz University for Health Sciences, Riyadh, Saudi Arabia

**Keywords:** Hepatitis C virus, Genotype, Blood donors, Viral load, Liver enzyme

## Abstract

**Background:**

Hepatitis C virus (HCV) genotypes and subtypes are considered an important tool for epidemiological and clinical studies and valuable markers for disease progression and response to antiviral therapy. The aim of this study was to identify the prevalence of HCV genotypes and their relation to socio-demographic factors particularly age and sex, various biochemical profiles and viral load.

**Methods:**

The records (630) of Saudi patients positive for HCV (2007–2011) reported in the system of the Molecular Pathology Laboratory at a tertiary reference hospital in Riyadh, Saudi Arabia were analyzed. Socio-demographic characteristics, liver biochemical profile, viral load and co-infection with HBV and HIV were retrieved from the hospital database. The associations of continuous and categorical variables with genotypes were analyzed.

**Result:**

The overall mean age of the surveyed patients was 59 years ±0.5 years (21% were <50 years (*p* = 0.02). The rate of infection is lower in males than in females (47.6% vs. 52.4%).

HCV genotype 4 was the most prevalent (60.7%), followed by genotype 1 (24.8%). However, genotype 1 and 3 were found more in males (29.7% vs. 20.3% and 6% vs. 2.1%, respectively, *p* = 0.001), while genotype 2 and 4 were more among females (4.8% vs. 2% and 68.5% vs. 52.3%, respectively). In addition, genotype 1 was found dominant in younger males (33.8%).

Biochemical parameters across gender showed significant variation in particular for the ALT (*p* = 0.007). The mean viral load was significantly higher in genotype 1 than genotype 4 (4,757,532 vs. 1,435,012, p = <001). There is a very low overall percentage of co-infection of HBV or HIV in this study (around 2% for each).

**Conclusion:**

Although HCV genotype 4 shows an overall high prevalence in this study, a clear decline in the rate of this genotype was also demonstrated in particular among the younger age group who displayed increasing trends toward the global trend of genotype 1, rather than genotype 4. This finding would be of clinical interest in relation to future planning of the therapy for HCV infected patient.

## Background

Hepatitis C virus (HCV) is a positive-strand RNA virus of the genus Hepacivirus within the Flaviviridae family infection [1]. It is a well-known causative agent of a serious, contagious, and inflammatory disease, which affects the normal function of the liver, predominantly as chronic infection [2, 3]. The global pattern of this infection is on the increase despite the improvement in transfusion practices and general health measures directed to limit the transmission of this disease [4].

Most studies have indicated that HCV is an endemic medical condition, and numerous steps are followed to eliminate the risk of this infection. Nonetheless the distributions of the variables of the viral infection such as genotype, age, and race across the world are on the increase despite the availability and efficacy of the HCV therapy [5, 6].

Indeed, various epidemiological studies have presented evidence indicating that the high incidence rate of HCV infection is a major threat for the population [5], and reports on the prevalence of HCV in Saudi Arabia are still inconsistent between different studies with wide variations in their target population. Although the prevalence of HCV was found to be around 50% among infected hemodialysis patients, the prevalence rates of 0.4–1.1% could be accepted among the general population based on data from blood donor screening centers [7–9].

On the other hand, the most prevalent genotype in Saudi Arabia is genotype 4, which is often followed by the genotype 1 [10–23]. These two genotypes are not only difficult to treat, but also are highly pathogenic. Most of these early studies in Saudi Arabia barely discussed the relation between the genotypes and age of the affected individuals. For example, the incidence of the infection was found to be high among the old people (>50 years) indicating past infection [24]. In most of those early publications the dominant genotype was genotype 4. This raised the question, whether the same genotype is currently prevalent in the younger Saudi generations? Also, could we predict the future genotype based on the currently available genotype analysis, in Saudi Arabia? Thus, the rationale behind conducting this study is to assess the relationship between HCV genotypes and certain epidemiologic and biologic features of HCV infected patients. Accordingly, the aim of this study was to find answers to these questions and also to correlate the HCV genotypes with the biochemical profiles and viral load. The study also aims to describe the prevalence of HCV genotypes based on socio-demographic factors (particularly age and sex) and to identify HCV genotypes with co-infection with HBV and HIV.

## Methods

### Participants and samples

A total of 630 Saudi patients were studied at a tertiary reference hospital in Riyadh, Saudi Arabia (King Abdulaziz Medical City). They were confirmed as HCV positive with the HCV-RNA genotype amplification polymerase chain reaction (PCR) method in the period from 2007 to 2011. HCV genotype results were retrieved from the Molecular Pathology Laboratory (MPL) database with additional information related to socio-demographic characteristics, and liver biochemical profile. The viral load (IU/ml) of the diagnosed patients was also retrieved from the Laboratory Information System (LIS) and QuadraMed Clinical Pathology Record (QCPR) system. Patients with incomplete HCV genotype results were excluded.

The recorded variables included: age, sex, clinical diagnosis, genotype, and biochemical profile, mainly alanine transaminase (ALT), aspartate aminotransaminase (AST), alkaline phosphates (ALP) and total serum bilirubin measures. Other variables related to the HCV characteristics were recorded, including viral load [reported in international units per milliliter (IU/mL) and copies per milliliter] and co-infection with HBV or HIV.

### Statistical analysis

The results are expressed as means ± standard deviations or as percentages. The means between groups were compared by using the student t test. The frequency distributions of the different genotypes within age groups and proportion were used. The associations between categorical variables were assessed using the Chi Square and Fisher’s exact tests. Statistical analysis was performed using SPSS 20.0 software (SPSS Inc., USA) and *P* values of 0.05 or less were considered significant.

No informed consent was needed since the analyzed laboratory data was secondary (without names or identity). Nonetheless, this study was reviewed and approved by the internal Institutional Review Board (IRB) in the College of Public Health and Health Informatics (CPHHI), King Saud bin Abdulaziz University for Health Sciences (KSAU-HS) and King Abdullah International Medical Research Center (KAIMRC) under # SP 14/053.

## Results

### Demographic characteristics (Table 1 and Table 2)

Socio-demographic factors of the patients’ positive for HCV genotypes are presented in Table 1. The mean age of the studied population was 59 years ±0.5 years. However, of the 630 recruited patients, 132 (21%) were below 50 years, and more than half (56.0%) were in the age group 50–69 years; the difference between the age groups is significant (*p* = 0.02). The rate of infection in male is lower than in females (47.6% vs. 52.4%, p = 0.02).

**Table 1:** Demographic Characteristics of the Study Group

Characteristics	Male		Female		Total	
Age (years) (μ = ± SD) 59 years ±0.5						
Age-groups (years) 59 ± 0.5 years (years)	No.	%	No.	%	No.	%
≤24	7	2.3	1	0.3	8	1.3
25–49	67	22.3	57	17.3	124	19.7
50–69	147	49.0	206	62.4	353	56.0
≥70	79	26.3	66	20.0	145	23.0
Total	300	47.6	330	52.4	630	100.0

*P* value <0. 020

**Table 2:** Distribution of genotype tests over a five year period (2007–2011)

Years	Male		Female		Total	
	No.	%	No.	%	No.	%
2007	37	12.30	37	11.2	74	11.7
2008	23	7.7	16	4.8	39	6.2
2009	84	28.0	86	26.1	170	27.0
2010	93	31.0	106	32.1	199	31.6
2011	63	21.0	85	25.8	148	23.5

*P* value <0. 409

The number of HCV infected cases increased gradually over the period of 5 years (2007–2011) (Table 2). The highest reported rate was in the year 2010 (31.6%) and the lowest in the year 2008 (6.2%), but the annual infection rate remained almost unchanged (*p* = 0.409).

### Distribution of genotypes according to gender and age (Table 3)

The overall distribution of the genotypes showed predominance of genotype 4 (60.7%) followed by genotype 1 (24.8%) including subtypes (a) and (b); for other genotypes the rate ranged between 3.5% for genotypes 2, 3 and mixed genotypes (3.5%, 4.1% and 7%, respectively). On the other hand, the genotype distribution varied significantly by gender (*P* < 0.001) as genotype 1 and 3 were more predominant in males, compared to females (29.7% vs. 20.3% and 6% vs. 2.1%, respectively, *P* = 0.001), while genotypes 2 and 4 were more predominant in females than in males (4.8% vs. 2% and 68.5% vs. 52.3%, respectively).

**Table 3:** The HCV genotype and subtype distribution are related to the genders

Characteristics	Male		Female		Total	
	No.	%	No.	%	No.	%
Genotype 1	89	29.7	67	20.3	156	24.8
Genotype 2	6	2	16	4.8	22	3.5
Genotype 3	18	6	7	2.1	25	4
Genotype 4	157	52.3	226	68.5	383	60.7
Mixed	30	10.0	14	4.3	44	7

*P* value <0.001

**Fig. 1:**
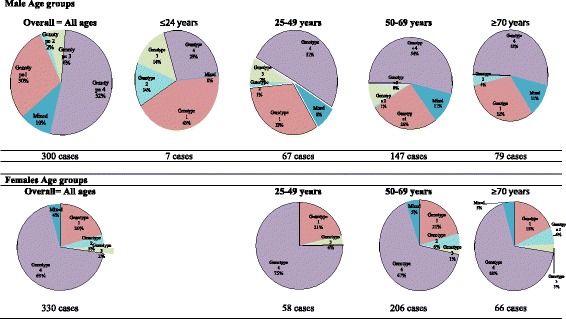
Genotypes distribution by age groups and gender

When comparing genotype 1 and 4 by age groups among the males and females, the overall rate of genotype 1 was higher among males than in females (30% vs. 20%, respectively, *P* < 0.001). Although a limited number of males was noted at the age group <50 years (74/300) with hepatitis C virus genotypes, 33.8% of them were males with genotype 1 infection, in comparison to females in the same age group (58/330, *P* < 0.001) seen with genotype 1 infection (20.7%). In contrast, genotype 4 was the predominant genotype among elder females (50–69 and ≥70 years) than among males of the same age groups (67% and 68%, vs 54% and 53%, respectively, *P* = 0.539). No significant differences were found in age between genotypes 1 or 4 (Fig. 1).

### Biochemical profile (Table 4 and Table 5)

Table 4 shows a statistically significant variation in the mean value of the biochemical parameters across gender of some biochemical parameters, in particular the ALT (*P* = 0.007); similar trend was also noted for the viral load (*P* < 0.001). No significant differences were found between gender with respect to the biochemical parameters such as AST, ALP and total serum bilirubin.

**Table 4:** Biochemical profile of the study group

Biochemical Profile	Male		Female		Total		*P*-value
Mean (±SD)^a^	Mean	±SD	Mean	±SD	Mean	±SD	
Viral Load^b^	3,460,339	7,231,276	1,615,937	3,946,460	2,499,596	228,849	<0.001
ALT	84.3	229.4	49.7	38.8	66.1	6.3	0.007
AST	93.2	303.6	177.4	2176.2	135 .5	61.7	0.507
ALP	128.8	97.1	120.1	81.3	124.5	3.5	0.223
Total serum Bilirubin	26.3	58.9	21.6	56.8	24.2	2.3	0.302

^a^ *SD* standard deviation; ^b^Viral Load expressed as international unit per milliliter (IU/ML). *ALT* Alanine transaminase, *AST* Aspartate aminotransaminase, *ALP* Alkaline phosphates

**Table 5:** HCV Genotype one and four Distribution across Biochemical Profile (630)

Biochemical Profile	Genotype	Mean	±SD	95% CI	
				Lower	Upper
Viral Load	1	4757532*	9,203,688	3,301,899	6,213,165
	4	1,435,012	3,047,317	1,128,855	1,741,169
ALT	1	57	59	48	66
	4	68	200	48	88
AST	1	81	293	35	127
	4	176	2028	−28	380
ALP	1	125	83	112	139
	4	123	95	114	133
Total Bilirubin	1	21	37	15	26
	4	25	60	18	31
Co-Infection (HBV/HIV**)**	1	3	1.8	–	–
	4	8	2.1	–	–

*SD* standard deviation, *CI* Confidence Interval; Genotype one (*n* = 156) and Genotype Four (*n* = 383); *ALT* Alanine transaminase, *AST* Aspartate aminotransaminase, *ALP* Alkaline phosphates *HBV* Hepatitis B virus, *HIV* Human Immunodeficiency Virus

*p value = <0.001

When focusing on the main issue of the current study, i.e. genotype 4 and genotype 1, the findings of the viral load showed significantly higher mean viral load in genotype 1 than genotype 4 (4,757,532 IU/ml vs. 1,435,012 IU/ml, *P* = <001). The rest of the tests showed no significant difference between genotype 1 and 4 (Table 5). As to the co-infection of HCV with either HBV or HIV, no significant relation could be established. Besides there is a very low overall percentage of co-infection in this study (around 2% for each).

Predictive factor analysis was conducted for genotype 1 using univariate and multivariate logistic analysis, considering genotype 4 as reference. The unadjusted odds of genotype 1 increased by 85.5% in males, 80% among patients with moderate viremia, 3 times more in patients with high viremia, and 90.7% among patients with high AST. However the odds decreased by 41.4% in patients with high ALT. According to the multivariate logistic regression analysis, the adjusted odds for genotype 1 showed an increase by 69.7% in males, 83.6% of patients with moderate viremia, and increased 3 times in patients with high viremia (Table 6).

**Table 6:** Univariate and multivariate analysis of gender, age, and biochemical profile in reference to genotypes 1 and 4^a^

Variables	OR	95% CI	*P* value	AOR	95% CI	*P* value
Age (reference <50 years)	0.976	0.615–1.547	0.916	–	–	–
Gender (reference female)	1.858	1.243–2.777	0.003	1.697	1.152–2.499	0.007
Moderate viremia (Low viremia reference)	1.800	1.119–2.893	0.015	1.836	1.149–2.934	0.011
High viremia (Low viremia reference)	3.157	1.897–5.253	0.000	3.116	1.887–5.144	0.001
AST (normal level as reference)	1.907	1.172–3.104	0.009	–	–	–
ALT (normal level as reference)	0.586	0.365–0.942	0.027	–	–	–
ALP (normal level as reference)	0.949	0.565–1.593	0.842	–	–	–
Total Bilirubin (normal level as reference)	1.026	0.625–1.684	0.920	–	–	–

^a^Genotype 4 as reference; *OR* unadjusted odds ratio, *AOR* adjusted odds ratio, *CI* Confidence Interval, *AST* Aspartate aminotransaminase, *ALT* Alanine transaminase, *ALP* Alkaline phosphates

Viremia or viral load is usually interpreted with magnitude of the virus in international unit per each milliliter of blood. Thus, it could be classified as low Viremia with magnitude of <200.000 IU/ml; moderate Viremia with the range from 200.000 IU/ml to 2000.000 IU/ml; and high Viremia with amount higher than 2000.000 IU/ml [58]

## Discussion

Hepatitis C virus is one of the most enduring causes of viral hepatitis, and numerous studies found strong connection between hepatitis C infection and various other infections, co-morbidities, and increased mortality rates [25].

The present study on the genotypes of 630 hepatitis C infected patients revealed that genotype 4 was confirmed in the majority of these patients (60.8%). This finding confirms previous reports where genotype 4 is a landmark of the infected cases of HCV in Saudi Arabia [7, 17, 18]. Besides, many review articles that described the global distribution of HCV genotypes [11, 23, 26, 27], defined clearly that genotype 4 is the dominant genotype in Middle Eastern countries, particularly Saudi Arabia, Egypt, Jordan, Yemen, Oman, and Sudan [11–13, 28]. We also found that genotype 4 was found in higher rate among females than males, but the different was not statistically significant, and this is in line with a similar earlier study in the Kingdom of Saudi Arabia (KSA) [12].

### Age and past exposure

In the present study 79% of HCV cases were above 50 years old, indicating that the majority of these cases were born before the year 1965. Past evidence showed that in Saudi Arabia, like other neighboring countries, most of the health and educational indicators did not reflect a satisfactory level of protection from exposure to blood borne contaminants including viral hepatitis [29, 30]. Such protective measures include among others, the unsafe and unprotected blood transfusion or the use of such phlebotomy procedure as one of the most common invasive procedures in health care for either the collection of blood or the injection of patients with medications. It was common to see a phlebotomist carry out such procedures known to increase the risk of needle-stick injury and transmission of blood borne infective microorganisms include viruses [9, 31, 32].

### Future prediction of HCV genotype 1

In Saudi Arabia as in most countries in the region, blood transfusion is quite recent therapeutic measure [33]. Nowadays, it is mandatory to undertake screening for the two major hepatitis viruses (HCV and HBV) in all donated blood in the country, and this includes testing for HCV (anti-HCV HCV RNA) and HBV (anti-HBc as well as the HBV RNA using molecular techniques) [16, 20]. Based on our assumption on HCV infection being more common disease among the old generations and with predominance of genotype 4, this status is certainly changing in the near future. There will probably be a tremendous decline in HCV infection rate, particularly the rate of genotype 4 but with dominance of genotype 1, thereby conforming with worldwide distribution of this genotype [26, 27]. Some studies have shown a clear decline in the incidence of HCV infection in the Saudi Arabia in comparison with previous data from the year 1990, with more decline among children compared to adults [9, 34]. This was explained by the fact that the perinatal and childhood transmission was not a major mode of transmission as assumed before the last three decades [34]. Blood transfusion is safer today than previously and is no longer a risk for HCV, HBV, or HIV transmission in the region, particularly after the improvements in the quality of blood supply with especial reliable screening tests and consequent reduction in the risk of transfusion-transmitted infectious diseases [26, 35, 36]. However, despite the availability of effective measures to ensure the quality and safety of blood and blood products, recently, the WHO has urged regional member states to ensure transfusion safety by promoting safe blood donation, reduce the risk associated with the clinical use, injection safety and infection control in the health care setting, and reducing the resort for unnecessary injections [37]. Issues concerning the safety of blood during the past 25 years have been associated with changes in blood use and triggered reevaluation of the clinical practices of blood transfusion [36]. Moreover, the role of health education and the high average income probably allowed more and better access to educational resources in preventive care including physician advice; all together played an important role in the reduction in the transmission of infection [38].

Although some early studies explored the dominance of HCV genotypes 4 in Saudi Arabia, the majority of these studies did not show the differences in genotypes according to age, Saudis versus non-Saudis, or other ethnicity related factors [7, 17, 18, 39, 40]. A recent study from KSA, showed increased trend of HCV genotype 1 among those below 50 years old in comparison to genotype 4 in the same age group (46.4% vs. 44.8%) [12]. Interestingly reports from Bahrain and the United Arab Emirates found genotype 1 to be dominant among the studied population [10, 41–43]. It is known that different genotypes of hepatitis C virus predominate in different regions of the globe. HCV genotypes 1, 2, and 3 can be found across the globe, while genotype 4 is mainly found in the Middle Eastern or African countries [7, 44, 45]. Confirming this observation, the present study found that 60% of the selected participants had genotype 4 HCV.

The current study has also compared the relationship between HCV genotypes and viral load (viremia level). Viral load refers to the number of virus particles present in the serum or blood. In this study, male patients demonstrated higher rates of viral load than female patients and that viral load was three times more in genotype 1 than genotype 4. Our findings were disagree with those of Al Zayed et al. where viral load levels (IU/ml) were more found with higher reading among patients with genotype 4; however, this could be due to the fact that this sample size was much smaller than our study [12]. Besides, our findings also disagreement from Indian results which showed that the viral load was higher in genotype 1 indicating that viral clearance of the virus was also delayed. This probably exacerbates the occurrence of higher rates of complications and chronicity of liver disease among their population [46, 47].

On the other hand the biochemical profiles showed a significant association of AST with genotype 1 and ALT with genotype 4. Clearly, the interaction between the virus and these enzyme in such population infected with HCV genotype 1 in particular needs more clarification and further definitions in large prospective studies.

Although, the rate of co-infections with HBV or HIV in the present study was very low in concordance with the genotypes, our findings demonstrated that patients infected with HCV genotype 4 did not carry any co-infection. This raises the question whether the genotype of HCV is a determinant factor of co-infection with other types of hepatitis viruses and/or with HIV for example? Further studies are needed on the association of other hepatitis viral infections with the specific genotype of HCV.

### Factors related to blood transfusion practices:

In Saudi Arabia, blood supply has shifted dramatically from imported blood, to paid donors and, lately, to the current total dependence on the indigenous predominantly voluntary donators with some contribution from donors mainly family members of patients [9]. However, some studies showed that the rate of blood donation among Saudis was less than satisfactory, probably due to misconceptions, poor knowledge and unfavorable attitudes towards donation [48–52].

As to the possible transmission of some of these transfusion-transmitted infectious diseases some of the patients in the present study, in the mid 1970–1990, must have received blood products mainly in the form of imported blood products which could have been contaminated by some of the viral agents including HCV before the era of the introduction of sensitive screening laboratory tests.

In his recent study on the epidemic dynamics of HCV Al-Qahtani has demonstrated clearly that genotype 4 was transmitted between countries in the region but originated from Egypt. Viral spread between Saudi Arabia and Egypt is predominantly directed towards Saudi Arabia, showing that both HCV genotype 4 epidemics are connected through a source-sink relation, perhaps linked to the large flow of Egyptian migrant workers [53].

Most of the studies including those by the WHO reported HCV prevalence in Saudi Arabia in the range of 0.4 to 1.1% [24, 54–56], which means that the rate of the infection categorizes the country among those with the lowest prevalence of HCV in the region, according to WHO classification [57].

This overall reduction in exposure rate may be due to several factors including public awareness of the disease, more and better access to health educational resources such as internet as well as preventive health care. Moreover, the improvement of socio-economic conditions and the level of general education might have also changed the social behavior and cultural habits resulting in reduction in the risks of contamination, in addition to the improvement in donor screening including the use of highly sensitive laboratory assays [38, 39].

Other measures have been undertaken by the government, such as the barring expatriate who are HCV carrier or chronic HBV carriers when applying for jobs in the KSA. No doubt these steps helped in limiting the sources of infection in the country^34^. Besides, the successful and effective therapy of chronic hepatitis C patients is now available in the KSA and this step helped in removing the residual sources of new infections. All these steps are expected to play a major contributory role in future in the transformation of the dominant genotype in the country^40^.

Converted rates of HCV genotypes were demonstrated in our study among the young and young adult generation (< 50 years old). This is probably due to the increased risk of being infected with the HCV genotype 1 among the non-Saudi residents in whom this genotype is dominant in their home countries. A study by Bashawri et al., (2004) found a higher rate of HCV genotype 1 among non-Saudi blood donors than Saudi donors^36^.

Nowadays, it is crucial to have detailed understanding of the relative HCV genotype prevalence and subtypes in the country and also to develop national treatment strategies including direct acting antiviral therapies. The outcome of such strategies will also facilitate undertaking therapeutic programs that will eventually show a clear cut-off point for the required duration of treatment, cure rates, and the need for interferon and other HCV anti-drugs.

According to our knowledge this is the first study of its types in Saudi Arabia on the correlation between the different HCV genotypes and their contribution to the liver and viral biomarkers.

## Conclusion

Although HCV Genotype 4 shows an overall high prevalence in the current cohort of the infected Saudi patients, a clear decline in the rate of this genotype was also demonstrated in particular among the younger age group. This is taken to indicate a trend in the transformation in the mapping of HCV genotypes towards the globalize dominance of genotype 1. This situation is not unique for Saudi Arabia but probably so in many other Arabic Gulf countries. The findings of this study could be of great value for those in clinical practice encouraging them to undertake the quantifying of the viral load before designing strategies for therapies in patients suffering from HCV infection. Further broad-based and age-stratified studies should be carried out to determine the future genotype mapping of HCV across the country.

## Abbreviations

ALP: Alkaline phosphates
ALT: Alanine transaminase
AST: Aspartate amino transaminase
CPHHI: College of Public Health and Health Informatics
HBV: Hepatitis B virus
HCV: Hepatitis C virus
HIV: Human Immunodeficiency virus
IRB: Institutional Review Board
IU/ml: International Unit per milliliter
KAIMRC: King Abdullah International Medical Research Center
KSA: Kingdom of Saudi Arabia
KSAU-HS: King Saud bin Abdulaziz University for Health Sciences
LIS: Laboratory Information System
MPL: Molecular Pathology Laboratory
PCR: Polymerase chain reaction
QCPR: QuadraMed Clinical Pathology Record
TB: Total bilirubin
